# DNA Methylation Changes Are Associated With an Incremental Ascent to High Altitude

**DOI:** 10.3389/fgene.2019.01062

**Published:** 2019-10-29

**Authors:** Ainash Childebayeva, Taylor Harman, Julien Weinstein, Jaclyn M. Goodrich, Dana C. Dolinoy, Trevor A. Day, Abigail W. Bigham, Tom D. Brutsaert

**Affiliations:** ^1^Department of Anthropology, University of Michigan, Ann Arbor, MI, United States; ^2^Department of Environmental Health Sciences, School of Public Health, University of Michigan, Ann Arbor, MI, United States; ^3^Department of Archaeogenetics, Max Planck Institute for the Science of Human History, Jena, Germany; ^4^Department of Exercise Science, Syracuse University, Syracuse, NY, United States; ^5^Department of Nutritional Sciences, School of Public Health, University of Michigan, Ann Arbor, MI, United States; ^6^Department of Biology, Faculty of Science and Technology, Mount Royal University, Calgary, AB, Canada; ^7^Department of Anthropology, University of California, Los Angeles, CA, United States

**Keywords:** altitude, epigenetics, DNA methylation, hypoxia, incremental ascent

## Abstract

Genetic and nongenetic factors are involved in the individual ability to physiologically acclimatize to high-altitude hypoxia through processes that include increased heart rate and ventilation. High-altitude acclimatization is thought to have a genetic component, yet it is unclear if other factors, such as epigenetic gene regulation, are involved in acclimatization to high-altitude hypoxia in nonacclimatized individuals. We collected saliva samples from a group of healthy adults of European ancestry (n = 21) in Kathmandu (1,400 m; baseline) and three altitudes during a trek to the Everest Base Camp: Namche (3,440 m; day 3), Pheriche (4,240 m; day 7), and Gorak Shep (5,160 m; day 10). We used quantitative bisulfite pyrosequencing to determine changes in DNA methylation, a well-studied epigenetic marker, in LINE-1, *EPAS1*, *EPO*, *PPARa*, and *RXRa*. We found significantly lower DNA methylation between baseline (1,400 m) and high altitudes in LINE-1, *EPO* (at 4,240 m only), and *RXRa*. We found increased methylation in *EPAS1* (at 4,240 m only) and *PPARa*. We also found positive associations between *EPO* methylation and systolic blood pressure and *RXRa* methylation and hemoglobin. Our results show that incremental exposure to hypoxia can affect the epigenome. Changes to the epigenome, in turn, could underlie the process of altitude acclimatization.

## Introduction

More than 140 million people worldwide permanently live at high altitudes, and 40 million more visit altitudes above 2,500 m annually (Ward et al., 2000; Moore, 2001). Atmospheric oxygen partial pressure decreases with increasing altitude, and most individuals experience physiological changes in low-oxygen environments, including increased ventilation, increased red blood cell production, and increased heart rate (HR) (Houston and Riley, 1947). A combination of molecular- to the organismal-level changes occurs during high-altitude acclimatization (Sarkar et al., 2003).

The acute response to hypoxia (seconds to hours) involves changes in homeostatic regulation, whereas chronic acclimatization (hours to years) is characterized by gene expression changes in the carotid body, endothelial cells, and other tissues (Kourembanas et al., 1990; Wiener et al., 1996; Huey and Powell, 2000). One of main responders to decreasing levels of oxygen is the hypoxia-inducible factor 1 (HIF) pathway. HIF-1 consists of two subunits, oxygen-regulated HIF-1α, and constitutively expressed HIF-1β (Ivan et al., 2001). In normoxic conditions, HIF-1α is hydroxylated by HIF prolyl hydroxylase (EGLN) and destined for degradation by ubiquitination *via* the von Hippel–Lindau ubiquitin ligase (Ohh et al., 2000; Epstein et al., 2001). HIF-1α hydroxylation is decreased in hypoxic conditions allowing it to accumulate and dimerize with HIF-1β forming an active HIF-1 transcription factor in the nucleus (Semenza, 2007). HIF is involved in promoting angiogenesis, regulating erythropoiesis, stimulating glycolysis, and inhibiting fatty acid oxidation (Formenti et al., 2010; Haase, 2013; Huang et al., 2014).

Previous studies have shown that mRNA levels of genes involved in the HIF pathway change upon hypoxic exposure, including *HIF1A* and *ARNT* in rats and mice (Wiener et al., 1996), mRNA levels of the platelet-derived growth factor (*PDGF-B*) in human endothelial cells (Kourembanas et al., 1990), and dopamine D2 receptor (*D2R*) in rat carotid body (Huey and Powell, 2000). Given their ability to change upon exposure to environmental factors, epigenetic mechanisms have been hypothesized to play a role the hypoxic response (Brown and Rupert, 2014). Epigenetics refers to mitotically and, in some cases, meiotically heritable changes to gene expression that do not involve changes to DNA sequence and may be reversible (Wolffe and Guschin, 2000; Feil and Fraga, 2011). The most widely studied and best understood epigenetic modification is DNA methylation, an addition of a methyl group to the nucleotide cytosine in a cytosine-guanine dinucleotide (CpG) (Mohn and Schubeler, 2009; Lam et al., 2012). DNA methylation is most commonly associated with gene repression when located in promoter regions of genes (Klose and Bird, 2006).

Epigenetic modifications are known for their plasticity and ability to change based on the environmental conditions (Bollati and Baccarelli, 2010). Previous studies found associations between DNA methylation and pharmaceuticals, exercise, stress, and other exposures (Dolinoy, 2007; Dolinoy and Jirtle, 2008; Senut et al., 2012; Faulk et al., 2014; Non et al., 2016). Decreased oxygen levels are associated with increased production of reactive oxygen species (ROS) that are genotoxic and can affect DNA methylation and the posttranslational modifications to histone proteins (James et al., 2004; Niu et al., 2015). Moreover, epigenetic changes have been observed in cancer cells that are often hypoxic due to the lower oxygen availability of solid tumors (Shahrzad et al., 2007; Baxter et al., 2014). Here, we focused on DNA methylation and exposure to high-altitude hypoxia.

Epigenetic regulation has been studied in the context of high-altitude adaptation in Andeans and Ethiopians (Alkorta-Aranburu et al., 2012; Childebayeva et al., 2019). Despite this, epigenetic changes associated with acclimatization to high-altitude hypoxia are not well understood (Julian et al., 2014). To determine if short-term exposure to hypoxia affects the epigenome, we recruited individuals trekking to Everest Base Camp in the Nepal Himalaya. We collected saliva samples and various physiological measurements at four different altitudes: Kathmandu [1,400m; baseline (BL)], Namche (3,440m; day 3), Pheriche (4,240m; day 7), and Gorak Shep (5,160m; day 10).

We determined the DNA methylation status of the repetitive element LINE-1 and the hypoxia-associated genes *EPAS1*, *EPO*, *PPARa*, and *RXRa*. We chose LINE-1 as the marker of global methylation as it has been shown to have different methylation profiles at high compared to low altitude in multigenerational Andeans of Quechua ancestry (Childebayeva et al., 2019). We examined methylation at *EPAS1* as polymorphisms near this locus are associated with hemoglobin levels in Tibetans (Beall et al., 2010), *EPO* as it is involves in red blood cell production (Eckardt et al., 1992; Dame et al., 1998; Beall et al., 2010), and *PPARa* and *RXRa* as these hypoxia-associated genes are involved in lipid metabolism regulation (Keller et al., 1993; Chinetti et al., 2000) and *PPARA* is associated with adaptation in high-altitude populations in the Himalaya (Keller et al., 1993; Chinetti et al., 2000; Simonson et al., 2010; Horscroft et al., 2017). *RXRa* and *PPARa* form a heterodimer that is necessary for *PPARa* functioning (Chan and Wells, 2009). The aforementioned HIF pathway genes have been chosen due to previous evidence that methylation levels at these genes are associated hypoxia (Rossler et al., 2004; Lachance et al., 2014; Cortese et al., 2016).

## Materials and Methods

### Ethics and Participant Recruitment

This study abided by the Canadian Government Tri-Council policy on research ethics with human participants (TCPS2) and the Declaration of Helsinki, except for registration in a database. Ethical approval was received in advance through Mount Royal University Human Research Ethics Board (protocol 100012 and 101361), the Syracuse University Institutional Review Board (protocol 18-006), and the University of Michigan Institutional Review Board (HUM00141118) and harmonized with the Nepal Health Research Council (protocol 109-2017).

This study took place in May 2018 as part of a research expedition in the Khumbu Valley, Everest region of Nepal. We recruited 21 healthy, nonpregnant, nonlactating, nonsmokers between 19 and 52 years of age from a larger research expedition to Everest Base Camp in the Nepal Himalaya. All participants were recruited in Kathmandu *via* verbal communication and provided written and informed consent prior to voluntary participation in the study. Even though these participants were recruited as a part of a larger research expedition, the research questions and data collection reported here were planned *a priori* in advance. Participants either were all altitude naive or had an extended period since the last altitude experience (> 1year). All participants were of self-reported European descent to control for population effects on epigenetics. Participant characteristics can be found in **Table 1**.

**Table 1:** Participant characteristics and DNA methylation.

	Kathmandu (1,400m)	Namche (3,400m)	Pheriche (4,370m)	Gorak Shep (5,160m)
LINE-1	64.55 (3.05)	62.83 (3.41)**	63.40 (2.12)*	63.70 (3.63).
*EPAS1*	6.48 (1.19)	6.55 (1.09)	6.95 (1.38)*	6.90 (1.35)
*EPO*	72.78 (4.88)	71.79 (4.14)	69.57 (4.13)*	71.22 (3.98)
*PPARa*	13.68 (4.16)	14.92 (4.51)	15.49 (3.79)**	16.12 (4.09)***
*RXRa*	40.01 (11.75)	35.13 (12.82)*	32.44 (9.05)***	33.57 (12.98)**
Hemoglobin (g/dL)	132.20 (26.67)	146.32 (18.13)**	149.42 (16.84)**	149.63 (26.06) **
Body mass index	22.69 (2.52)	22.58 (2.46)	22.45 (2.36)**	22.25 (2.34)***
Systolic blood pressure (mmHg)	119.62 (12.29)	124.62 (10.60).	120.62 (10.91)	126.67 (17.98)*
Diastolic blood pressure (mmHg)	83.00 (6.61)	87.95 (7.61)**	87.19 (8.45)**	86.38 (9.01)*
Peripheral oxygen saturation	96.86 (1.11)	92.67 (3.26)***	89.29 (2.72)***	81.48 (4.80)***
% Female		45.45 (24.65, 66.26)		
Age (year)		24.41 (8.20)		

Data are means (SD) of average measurement per individual, 95% confidence interval (CI) for proportions in brackets. Age is presented as mean (SD).

Significance symbols denote the difference between Kathmandu baseline and each altitude.

.p<0.10. *p<0.05. **p<0.01. ***p<0.001.

### Ascent Profile and Data Collection

**Figure 1:**
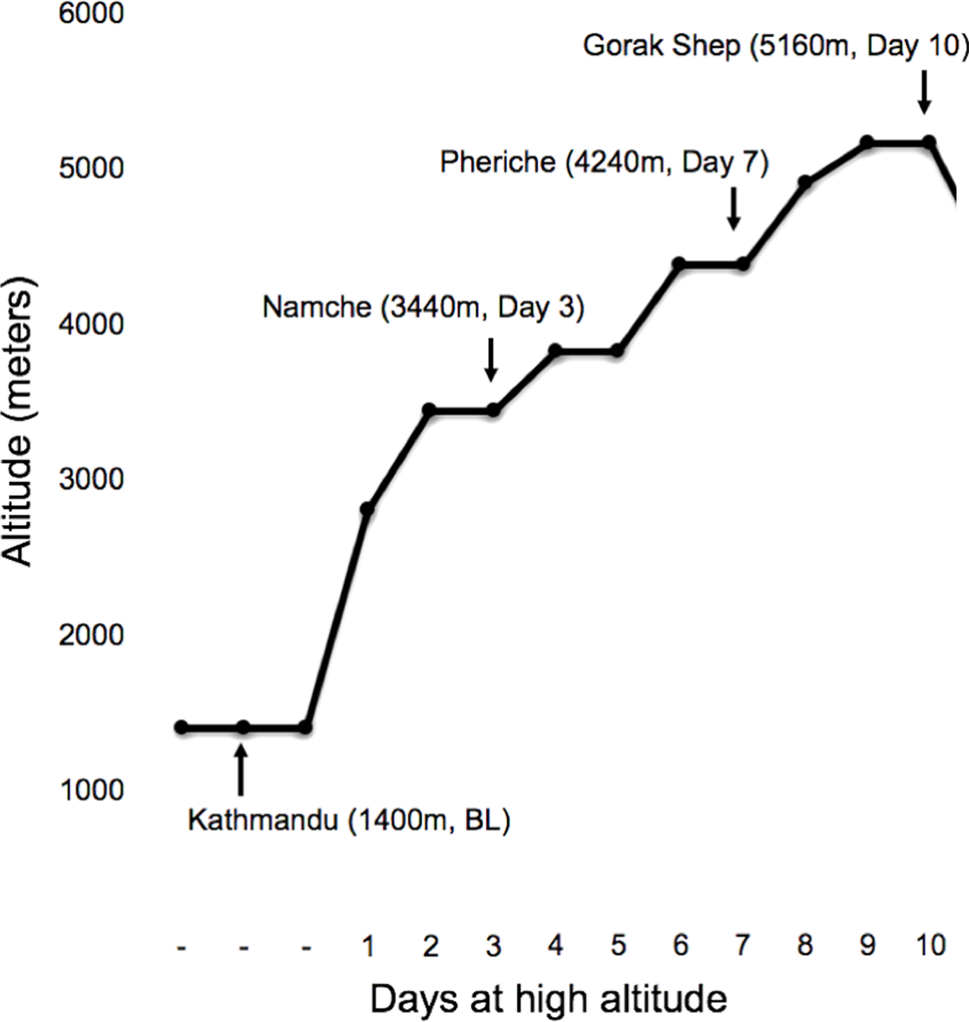
Trekking profile with the sampling locations marked by arrows.

Over the course of 10 days, a team of researchers and study participants trekked from 2,800 to 5,160 m. The ascent profile included three nontrekking rest days at 3,440 m (day 3), 3,820 m (day 5), and 4,240 m (day 7; **Figure 1**). In the morning between 6:00 and 8:00 local time at 1,400m (Kathmandu; day 0), 3,440 m (Namche; day 3), 4,240 m (Pheriche; day 7), and 5,160 m (Gorak Shep; day 10), saliva samples for DNA and physiological measures were taken following one night of sleep at each altitude.

With respect to physiological measures, body weight was measured using a portable digital scale (model HBF-516B; Omron, San Ramon, CA, United States). All physiological measures were obtained at rest in a seated position following >2-min rest with eyes closed and white noise played through headphones to limit distraction. Blood pressure was assessed using an automated sphygmomanometer. Peripheral oxygen saturation and HR (min^−1^) were measured using a portable finger pulse oximeter (Masimo SET Rad-5, Danderyd, Sweden). Hemoglobin concentration [(Hb); Hemocue Hemoglobin System, Hb201+, Angelholm, Sweden] was assessed *via* finger capillary blood sample using sterile lancets and universal precautions. Self-reported acute mountain sickness scores were obtained using the standard Lake Louise Questionnaire (Roach Rc et al., 1993). All phenotypic measures were performed at the same time of day for each participant. Physiological data can be found in **Table 1** and **Supplementary Table 1**.

### DNA Methylation

Saliva samples were collected, and DNA was extracted following a well-established protocol (Quinque et al., 2006). Quantitative pyrosequencing was performed to assess DNA methylation levels of LINE1, *EPAS1*, *EPO*, *PPARa*, and *RXRa*. Five hundred nanograms of DNA from each sample was bisulfite converted using the EZ-96 DNA Methylation^™^ Kit (Zymo Research, Irvine, CA, USA). Bisulfite-converted DNA was amplified using primers for LINE1 and each targeted gene and HotstarTaq plus Master Mix (Qiagen, Valencia, CA, USA). Primer sequences and the locations of the amplicons can be found in **Supplementary Table 2**. Each sample was pyrosequenced in duplicate using the Pyromark Q96 pyrosequencer (Qiagen). Quality control of the data was assessed using quality control measures built into the pyrosequencing software. All measurements outside of 2 standard deviations from the mean of all samples for each CpG position were excluded. Moreover, measurements with the coefficient of variance between replicates of more than 10 were excluded from further analyses. Duplicate measurements were averaged, as was DNA methylation at CpG sites within each gene. Statistical modeling was performed on these average DNA methylation values for each subject at each gene. Statistical analyses were performed using the samples collected from the 21 individuals at four altitudes for each LINE1, *EPAS1*, *EPO*, and *PPARa* and 19 for *RXRa*. No template controls and 0% methylated DNA and 100% methylated DNA controls (Qiagen) were included in all experiments.

### Statistical Analysis

We used R version 3.5.1 (R Core Team, 2018). Packages lme4 (Bates et al., 2015), lmerTest (Kuznetsova et al., 2017), ggplot2 (Wickham, 2009), and directlabels (Hocking, 2018) were employed in our statistical analysis and plotting. Linear mixed-effects modeling was used to account for replicate measurements at each altitude. The following linear mixed-effects models were tested. Study participants were modeled as random effects to account for repeated measurements. We included age and sex in the models, since both are known to affect DNA methylation (Liu et al., 2010; Hernandez et al., 2011; Lam et al., 2012; Horvath, 2013).

Yi (% methylation) ∼ B00 + B01(X) + B02(Sex) + B03(Age) + (1|ID) + ei, where *X* = low altitude (1,440m, BL) vs. high altitude [3,440m (day 3), vs. 4,240m (day 7), vs. 5,160m (day 10) combined], ID = sample ID.

Yi (% methylation) ∼ B00 + B01(X) + B02(Sex) + B03(Age) + (1|ID) + ei, where *X* = altitude [1,400 (BL) vs. 3,440m (day 3), vs. 4,240m (day 7), vs. 5,160m (day 10)], ID = sample ID.

Yi (% methylation) ∼ B00 + B01(X) + B02(Altitude) + B03(Sex) + B04(Age) + (1|ID) + ei, where *X* = phenotype, altitude = 1,400m (BL), 3,440m (day 3), 4,240m (day 7), 5,160m (day 10), ID = sample ID.

## Results

### Hypoxic Exposure Is Associated With Changes in DNA Methylation

**Figure 2:**
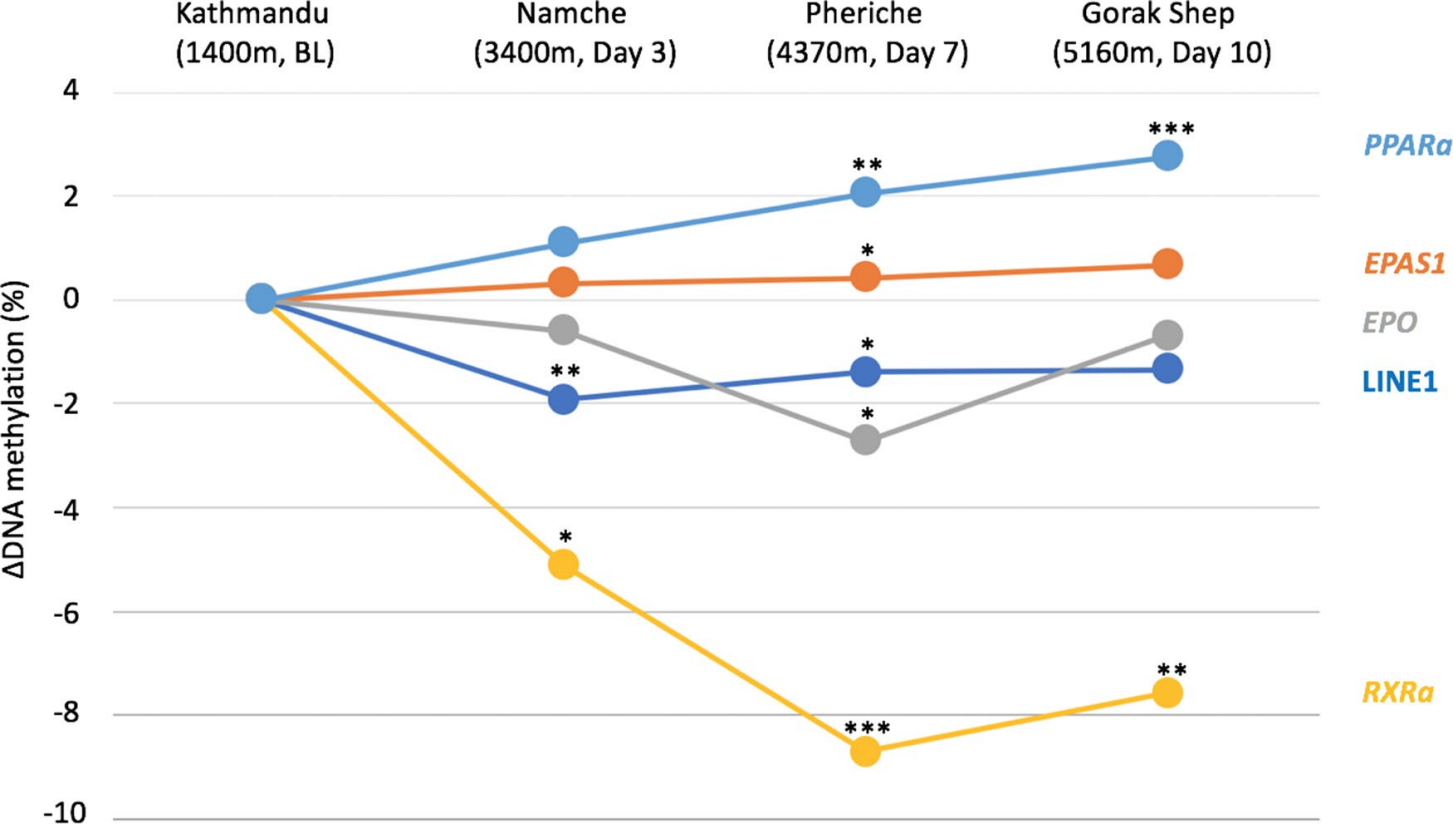
DNA methylation difference between Kathmandu and each altitude is plotted for each gene over time. The baseline is Kathmandu, which is 0. Significance levels are shown by *p<0.05, **p<0.005, ***p<0.001.

We found LINE1 methylation to be negatively associated with altitude when comparing low altitude 1,400 m (BL) to high altitude [3,440 m (day 3) + 4,240 m (day 7) + 5,160 m (day 10)] (β = −1.62 (high), *p* = 0.005) (**Table 2**, **Figure 2**, and **Supplementary Figure 1A**). Methylation levels of LINE1 were also significantly lower at 3,440 m (day 3) and 4,240 m (day 7) compared to 1,400 m (BL) (**Table 2**, **Figure 2** and **Supplementary Figure 1B**).

**Table 2:** Associations between DNA methylation and altitude.

		β	*p*
***LINE-1***			
	Low vs. high*	−1.62 (High)	0.005 **
	3,440m (day 3)**	−2.04	0.003 **
	4,240m (day 7)**	−1.47	0.037 *
	5,160m (day 10)**	−1.32	0.055.
***EPAS1***			
	Low vs. high*	0.36 (High)	0.096.
	3,440m (day 3)**	0.27	0.310
	4,240m (day 7)**	0.58	0.033*
	5,160m (day 10)**	0.22	0.393
***EPO***			
	Low vs. high*	−1.34 (High)	0.171
	3,440m (day 3)**	−0.61	0.603
	4,240m (day 7)**	−2.71	0.023 *
	5,160m (day 10)**	−0.69	0.553
***PPARa***			
	Low vs. high*	1.97 (High)	0.002 **
	3,440m (day 3)**	1.10	0.125
	4,240m (day 7)**	2.05	0.005 **
	5,160m (day 10)**	2.76	< 0.001***
***RXRA***			
	Low vs. high*	−7.14 (High)	< 0.001***
	3,440m (day 3)**	−5.13	0.039 *
	4,240m (day 7)**	−8.70	< 0.001***
	5,160m (day 10)**	−7.58	0.003 **

Low vs. high refers to 1,400m (BL) vs. 3,440, 4,240, and 5,160m combined. Otherwise, results indicate the difference between 1,400m (BL) and the altitude listed. All mixed-effects models were adjusted for age and sex.

.p<0.10. *p<0.05. **p<0.01. ***p<0.001.

The association between *EPAS1* methylation and high vs. low altitude approached significance (β = 0.36 (high), *p* = 0.096, **Supplementary Figure 1B**), and only the comparison between 1,400 m (BL) and 4,240m (day 7) was significant at *p*<0.05 (β = 0.58, *p* = 0.033, **Table 2**).

*EPO* methylation was not significantly different between high and low altitude [β = −1.34 (high), *p* = 0.171] (**Table 2**, **Figure 2** and **Supplementary Figure 1C**), and only the comparison between 1,400 and 4,240 m (day 7) was significant (β = −2.71, *p* = 0.023, **Table 2**, **Figure 2**).

*PPARa* methylation was positively associated with high altitude [β = 1.97 (high), *p* = 0.002, **Figure 2** and **Supplementary Figure 1D**], and the comparisons between 1,400 m (BL) vs. 4,240 m (day 7) and 5,160 m (day 10) were significant (**Table 2**).

We observed decreased methylation of *RXRa* associated with high altitude [β = −7.14 (high), *p*<0.001, **Figure 2** and **Supplementary Figure 1E**]. Moreover, *RXRa* methylation levels at 3,440 m (day 3), 4,240 m (day 7), and 5,160 m (day 10) were significantly lower than at 1,400 m (BL) (**Table 2**).

### Associations Between DNA Methylation and Phenotypic Data

Systolic blood pressure was positively associated with *EPO* methylation (β = 0.63, *p* = 0.022, **Supplementary Table 3**). This relationship was seen for baseline, day 3, and day 10 but not day 7 (**Supplementary Figure 2B**). We found a significant association between increased *RXRa* DNA methylation and increased hemoglobin levels (β = 0.54, *p* = 0.038, **Supplementary Table 3**). This general relationship was observed for each altitude, except the low-altitude baseline (**Supplementary Figure 2A**). We also identified associations approaching significance between *PPARa* methylation and hemoglobin, *RXRa* methylation and systolic blood pressure, body mass index, and *EPO*, and mean arterial pressure and *EPO* methylation (**Supplementary Table 3**). We did not find any significant associations between hemoglobin levels and *EPAS1* or *EPO* methylation (data not shown).

## Discussion

Oxygen homeostasis is an essential component of basic physiological homeostasis. In the mitochondria, oxygen is used to produce ATP in the process of oxidative phosphorylation (Semenza, 2007). HIF downregulates oxygen consumption by mitochondria and stimulates the glycolytic pathway enzymes (Semenza, 2010). In addition, the HIF pathway is involved in the regulation of fatty acid metabolism (Huang et al., 2014; Liu et al., 2014). Fatty acid oxidation is inhibited in hypoxic conditions as a result of the switch to glycolysis. This leads to accumulation of lipid droplets that have been shown to play a role in protection against ROS (Whitmer et al., 1978; Bensaad et al., 2014). Moreover, the HIF pathway is involved in increasing hemoglobin levels in response to decreased oxygen levels (Franke et al., 2013). In this study, we determined DNA methylation levels of four HIF pathway genes involved in oxygen homeostasis and metabolism, *EPAS1*, *EPO*, *PPARa*, and *RXRa*, and the marker of global methylation, LINE-1, to better understand how the epigenome responds to changes in ambient oxygen availability.

This is the first study to report changes in DNA methylation associated with an incremental ascent to high altitude in a cohort of European ancestry. Previous studies have shown that DNA methylation is affected by chronic exposure to hypoxia (Watson et al., 2010; Yuen et al., 2013; Brown and Rupert, 2014; Childebayeva et al., 2019). However, the effects of short-term hypoxic exposure on the epigenome have not been studied in the context of acclimatization to high altitude in nonacclimatized individuals.

We found decreased LINE-1 methylation, increased *PPARa*, and decreased *RXRa* methylation at high compared to low altitude. We also identified increased *EPAS1* methylation at 4,240 m (seven days of ascent) and decreased *EPO* methylation at 4,240 m compared to 1,400m. We also found positive associations between *RXRa* methylation and hemoglobin and between *EPO* methylation and systolic blood pressure. These findings show that short-term exposure to high-altitude hypoxia can influence the epigenome, which may in turn influence gene expression and phenotype and thus contribute to high-altitude acclimatization.

LINE-1 is a repetitive element, and its methylation level is associated with the global genomic methylation level (Ogino et al., 2008a; Iwagami et al., 2012). The methylation status of LINE-1 has been used as a proxy for the status of the methylome upon exposure to toxicants and in cancer (Chalitchagorn et al., 2004; Kile et al., 2012). Decreased LINE-1 methylation has been shown in cancer and has been associated with genomic instability (Ogino et al., 2008b; Pattamadilok et al., 2008). We found lower LINE-1 methylation levels associated with high-altitude exposure in our cohort. This could be explained by the effect of ROS on the genome, since ROS production is higher in hypoxic conditions (Wongpaiboonwattana et al., 2013; Kloypan et al., 2015). This finding likely reflects the effects of hypoxia as a stressor on the genome.

*EPAS1* is involved in activation of oxygen-regulated genes, plays a role in vascular remodeling (Peng et al., 2000), and is an important regulator of EPO, which controls erythropoiesis (Rankin et al., 2007). Importantly, *EPAS1* contributes to high-altitude adaptation in Tibetans and shows altered methylation in Andeans (Beall et al., 2010; Childebayeva et al., 2019). We found increased methylation of *EPAS1* associated with high altitude at 4,240m (day 7), potentially suggesting decreased *EPAS1* expression. In comparison, high-altitude adapted Andeans show decreased methylation in hypoxic conditions compared to normoxia (Childebayeva et al., 2019). It is possible that the increase in *EPAS1* methylation corresponds to the increase in *EPAS1* hydroxymethylation. Hydroxymethylation of a CpG site is an intermediate stage in the demethylation pathway catalyzed by the ten-eleven translocation (TET) family enzymes (Tahiliani et al., 2009). The bisulfite conversion method we used does not differentiate between methylated and hydroxymethylated cytosines (Nestor et al., 2010). It is possible that *EPAS1* is in the process of demethylation, which would be expected in hypoxic conditions, although we are seeing a general increase in DNA methylation in our participants.

*EPO* plays a major role in increased erythropoiesis under HIF control (Koury and Bondurant, 1990; Yoon et al., 2006; Risso et al., 2007). EPO levels rise quickly upon hypoxic exposure (Eckardt et al., 1989). In previous studies of high-altitude acclimatization, EPO has been shown to peak after 1 to 3 days at altitude, followed by a decline in an altitude-dependent manner (Abbrecht and Littell, 1972; Ge et al., 2002). EPO has a conserved HIF-1 binding site (HBS) CGTG in its 3′ UTR containing a CpG site (Wang and Semenza, 1993; Wang and Semenza, 1996; Wenger et al., 1998). Decreased methylation status of the HBS has been associated with activation of EPO in hypoxic conditions (Wenger et al., 1998). We found lower methylation upstream of the HBS site at all higher altitudes compared with 1,400m, and this was statistically significant at 4,240m. These findings are concordant with previously observed increased EPO expression levels at high altitude (Robach et al., 2004) and suggest that DNA methylation may play a role in this process.

We found a significant positive association between EPO methylation and systolic blood pressure suggesting that lower levels of erythropoietin may be correlated with higher blood pressure, since higher DNA methylation around this locus just upstream of an HBS has been linked to decreased expression of EPO based on previous research (Wenger et al., 1998). Other studies have shown a positive relationship between erythropoietin and human recombinant erythropoietin and blood pressure at rest and exercise in humans and in hypertensive and normotensive rats (Berglund and Ekblom, 1991; Raine and Roger, 1991; Muntzel et al., 1993). More research is necessary to establish if decreased EPO methylation of the locus targeted here is truly associated with higher levels of erythropoietin and if there is a link between *EPO* methylation and systolic blood pressure, especially since we did not find an association with hemoglobin. Of note, we observed a negative relationship between *EPO* methylation and systolic blood pressure at 4,240m (day 7), indicating a potential positive link between EPO expression and blood pressure at this altitude. This is the only altitude where we observed a significant change in methylation compared to the baseline. Further investigation is necessary to determine why the positive relationship between *EPO* expression and systolic blood pressure was identified only at 4,240 m of altitude (day 7).

*PPARa* is a transcription factor involved in controlling fatty acid metabolism and oxidation (Keller et al., 1993). PPARs activate the gene for acyl coenzyme A oxidase, which is the rate-limiting enzyme of the peroxisomal β-oxidation pathway (Dreyer et al., 1992; Keller et al., 1993). In addition to its role in fatty acid metabolism, *PPARa* is also associated with conditions such as obesity and diabetes, as well as various cardiovascular conditions including hypertension and atherosclerosis (Belanger et al., 2002). *PPARa* promotes fatty acid oxidation and may be involved in the switch from fatty acid oxidation to glucose oxidation *via* regulation of uncoupling protein 3 (Teruel et al., 2000; Gilde et al., 2003).

HIF transcription factors are known regulators of metabolism (Formenti et al., 2010). Previous studies in cell cultures have shown that *PPARa* is downregulated by HIF-1 in hypoxic conditions, which may be an adaptive response to hypoxia-induced inflammatory stimuli and metabolic changes (Narravula and Colgan, 2001). Another study of hypoxia exposure during an incremental ascent to the Everest Base Camp has found lower capacity for fatty acid oxidation in skeletal muscle and lower *PPARa* expression at altitude in the Himalayan Sherpa compared to lowlanders (Horscroft et al., 2017).

We found increased *PPARa* methylation associated with increasing altitude. The region we targeted is in the promoter region of *PPARa* suggesting that increased methylation here would be associated with a decrease in expression of *PPARa*, which is consistent with previous findings of decreased *PPARa* expression in hypoxic conditions (Narravula and Colgan, 2001). Decreased expression of *PPARa* is associated with diminished breakdown of fatty acids (Yoon, 2009). Lower levels of fatty acid oxidation are hypothesized to occur in hypoxic conditions due to the switch to anaerobic glycolysis (Ge et al., 2012).

*RXRa* is a transcription factor involved in fat metabolism and intracellular receptor signaling. *RXRa* binds to *PPARa* forming an active transcriptional complex able to bind to target genes known as proliferator-responsive elements (Dreyer et al., 1993). Several studies have shown that the activity of the PPARa/RXR*a* complex is reduced in hypoxic conditions to enable suppression of fatty acid metabolism (Huss et al., 2001; Belanger et al., 2007). The *RXRa* pathway was altered by hypobaric hypoxia exposure in the rat brain (Sethy et al., 2011). RXRs play a protective role in H9c2 cardiomyocytes from hypoxia/reoxygenation–induced oxidative injury in rats (Shan et al., 2014). We found decreased methylation of the CpG island located in the promoter region of *RXRa*, which may be associated with increased expression of *RXRa*.

Interestingly, we observed opposite trends in *PPARa* and *RXRa* methylation change. For example, individuals with increased *PPARa* methylation at high altitude have decreased *RXRa* methylation (**Supplementary Figures 1D, E**). Interestingly, individuals with decreased *PPARa* methylation (IDs 7 and 17) at high altitude have increased *RXRa* methylation (IDs 7 and 17), further highlighting the interactive nature of *PPARa/RXRa*. Since we see opposite change in *PPARa* and *RXRa* methylation levels, it is unclear whether it indicates increased or decreased activity of the PPARa/RXR*a* complex.

We found a significant positive association between *RXRa* methylation and hemoglobin levels. *RXRa* is a member of the retinoic acid receptor family and is necessary for normal hematopoiesis during development (Melnick and Licht, 1999; Oren et al., 2003). Retinoic acid signaling, specifically retinoic acid receptor α, is also involved in adult hematopoiesis (Canete et al., 2017). RXRa has been shown to play a role in hematopoietic signaling in mice (Ricote et al., 2006). However, the role of RXRa in this process is not well understood in adult humans. Our data suggest that there is a relationship between *RXRa* and hemoglobin levels. Previous studies have shown that *PPARa* is associated with hemoglobin levels, which could explain why we see a significant association with *RXRa* as well, since PPARa and RXRa are known to interact (Simonson et al., 2010; Scheinfeldt et al., 2012).

One of the limitations of our study is the use of saliva as a source of DNA. Saliva comprised white blood cells and epithelial buccal cells. Due to differences in DNA methylation signatures between tissues, any changes in methylation associated with altitude exposure may be confounded by cell type composition differences between altitudes (Lokk et al., 2014). We did not quantify saliva cell types at each altitude and thus were unable to control for this limitation. Furthermore, we only assessed the methylation levels of saliva. Therefore, it is unclear how other tissues responded to hypoxic exposure. However, there are known correlations between saliva and blood. Studies of genome-wide DNA methylation have reported 88.5% to 96.7% Pearson correlation between blood and saliva CpG sites within an individual (Thompson et al., 2013; Smith et al., 2015a). Saliva buccal epithelial cell methylation is also similar to the methylation patters of the brain due to the same ectodermal developmental origin and thus may serve as a proxy for DNA methylation changes in the brain (Smith et al., 2015b). Methylation levels of certain CpG sites are known to be tissue-specific, while methylation levels of other CpG sites correlate between tissue types (Varley et al., 2013). To our knowledge, the genes and loci we chose to study have not been shown to be tissue-specific in terms of methylation levels.

Lastly, we did not collect gene expression data from our participants and thus have not been able to directly link changes in DNA methylation to gene expression.

Overall, we found that short-term exposure to high-altitude hypoxia can affect the epigenome. We observed changes in LINE-1 and hypoxia-pathway associated genes *EPAS1*, *EPO*, *PPARa*, and *RXRa*. We also found significant associations between DNA methylation of EPO and RXRa and systolic blood pressure and hemoglobin, respectively. Our findings contribute to the growing literature on the role of epigenetics in acclimatization to high altitude. Future studies of the genome-wide effects of hypoxia on epigenetics are necessary to better understand the extent of DNA methylation change upon high-altitude exposure.

## Data Availability Statement

The datasets generated for this study are available on request to the corresponding author.

## Ethics Statement

This study abided by the Canadian Government Tri-Council policy on research ethics with human participants (TCPS2) and the Declaration of Helsinki, except for registration in a data base. Ethical approval was received in advance through Mount Royal University Human Research Ethics Board (Protocol 100012 and 101361), the Syracuse University Institutional Review Board (Protocol 18-006), the University of Michigan Institutional Review Board (HUM00141118) and harmonized with the Nepal Health Research Council (Protocol 109-2017). All participants were recruited *via* verbal communication and provided written and informed consent prior to voluntary participation in the study.

## Author Contributions

TB, AC, AB, TD, DD, and JG conceived and designed the research. AC, TH, and JW performed experiments. AC and TH analyzed data. TB, AC, and TH wrote the manuscript with contributions from all authors.

## Funding

This work was supported by the University of Michigan (to AC) and the Michigan Lifestage Environmental Exposures and Disease (M-LEEaD) National Institute of Environmental Health Sciences Core Center (P30 ES017885 to DD). AC was supported by the Marshall Weinberg Award and the National Geographic Early Career Award (EC-50834R-18). TD was supported by a Natural Sciences and Engineering Research Council of Canada Discovery grant (RGPIN-2016-04915).

## Conflict of Interest

The authors declare that the research was conducted in the absence of any commercial or financial relationships that could be construed as a potential conflict of interest.
