# Supplementary material for: DNA Methylation Changes Are Associated With an Incremental Ascent to High Altitude
Source: Front Genet. 2019 Oct 29;10:1062. doi: 10.3389/fgene.2019.01062 (PMC6828981; doi:10.3389/fgene.2019.01062)
Supplement: Supplementary file 2 [file Presentation_1.pptx]

## Slide 1
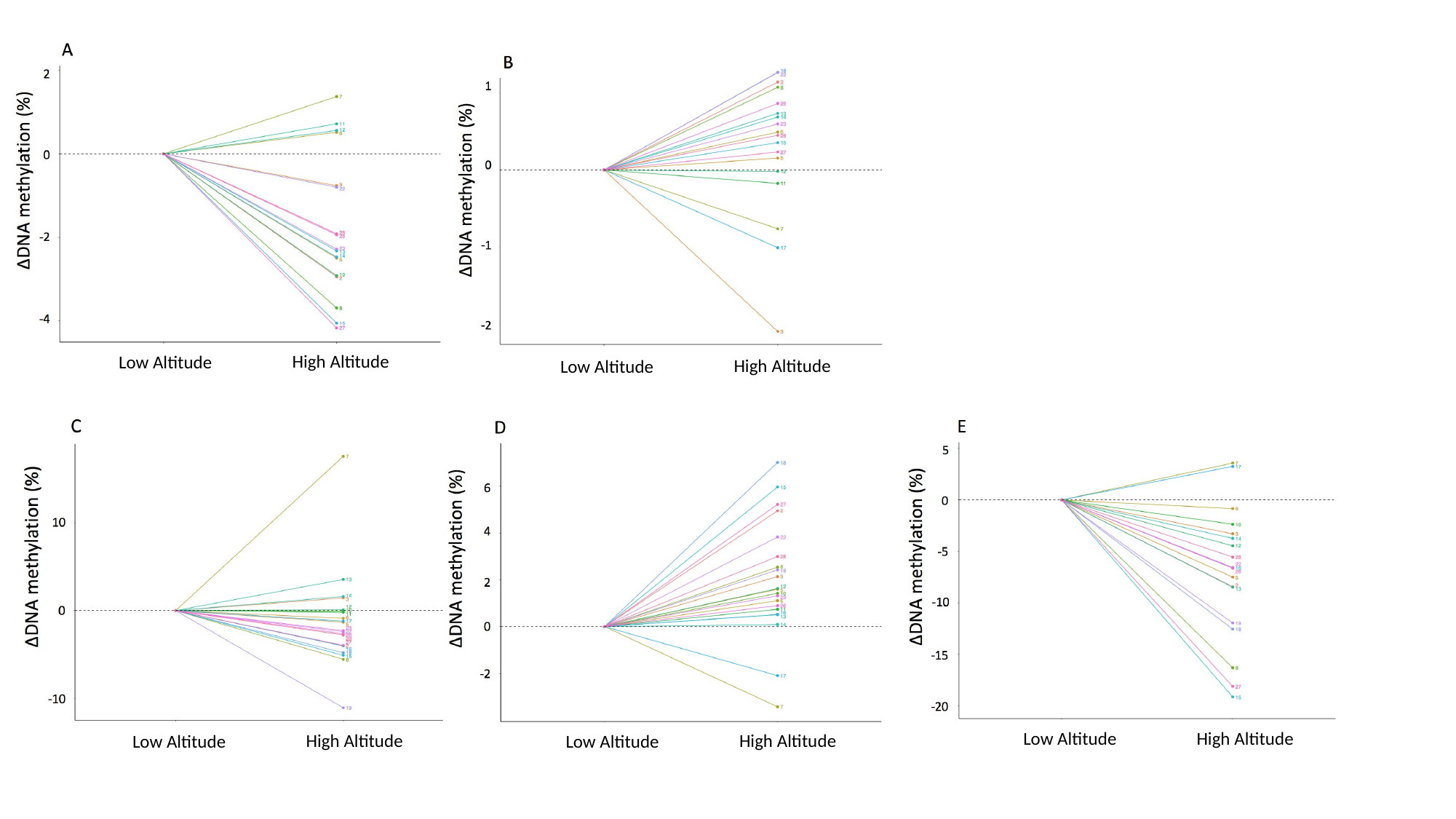

High Altitude
Low Altitude
High Altitude
Low Altitude
High Altitude
Low Altitude
High Altitude
Low Altitude
High Altitude
Low Altitude

## Slide 2
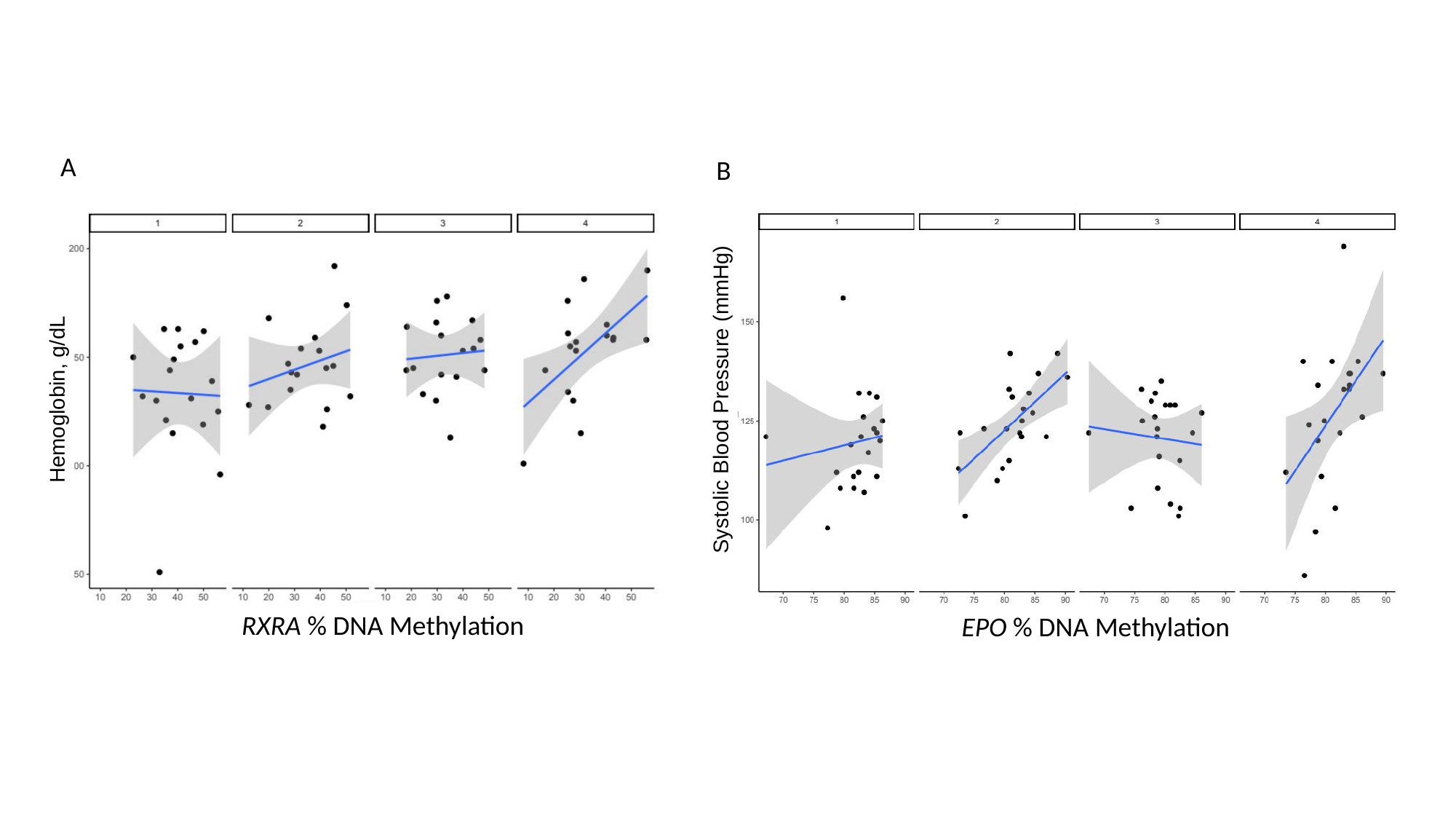

A
Hemoglobin, g/dL
RXRA % DNA Methylation
B
Systolic Blood Pressure (mmHg)
EPO % DNA Methylation
